# Neuropsychological Functioning and Coping Strategy Intervention Approaches in Youth with Posttraumatic Stress Disorder

**DOI:** 10.3390/medicina62050933

**Published:** 2026-05-11

**Authors:** Kalliopi Megari, Dimitra V. Katsarou, Georgios A. Kougioumtzis, Evangelos Mantsos, Maria Sofologi, Agathi Argyriadi, Alexandros Argyriadis, Efthymia Efthymiou

**Affiliations:** 1 Department of Psychology, University of York, Europe Campus, L. Sofou 3, 54621 Thessaloniki, Greece; 2 School of Social Sciences and Humanities, Department of Psychology, University of Western Macedonia, 53100 Florina, Greece; 3 Department of Preschool Education Sciences and Educational Design, University of the Aegean, 85132 Rhodes, Greece; d.katsarou@aegean.gr; 4 Department of Turkish Studies and Modern Asian Studies, Faculty of Economic and Political Sciences, National and Kapodistrian University of Athens, 15772 Athens, Greece; kougioum@uoa.gr; 5 Department of Occupational Therapy, University of Western Macedonia, 50200 Ptolemaida, Greece; evmantsos@uth.gr; 6 Department of Early Childhood Education, School of Education, University of Ioannina, 45110 Ioannina, Greece; m.sofologi@uoi.gr; 7 Department of Psychology, School of Arts and Sciences, American University in Dubai, Sheikh Zayed Road, Dubai P.O. Box 28282, United Arab Emirates; 8 Department of Nursing, Hellenic Mediterranean University, 71410 Heraklion, Greece; alexargiriadis@gmail.com; 9 Department of Interdisciplinary Studies, Zayed University, Abu Dhabi 144534, United Arab Emirates; efthymia.efthymiou@zu.ac.ae

**Keywords:** attention, child and adolescent, cognitive rehabilitation, executive function, emotion regulation, mindfulness, neurofeedback, neuroplasticity, trauma exposure, posttraumatic stress disorder, trauma-focused cognitive behavioral therapy

## Abstract

*Background*: Posttraumatic stress disorder (PTSD) in ages 3–18 is associated with disturbances in attention, working memory, processing speed, and executive control, as well as persistent difficulties in affect regulation. These neuropsychological vulnerabilities might interfere with learning, peer relationships, and the consolidation of age-appropriate developmental skills. *Methods*: We conducted a narrative review informed by a structured literature search in PubMed, Scopus, PsycINFO, Embase, EBSCOhost, Web of Science, and Google Scholar. English-language publications from 1990 to 2025 were considered if they examined (1) neuropsychological outcomes of trauma exposure or PTSD in youth and/or (2) interventions with potential to modify neurocognitive or affective functioning, including trauma-focused cognitive behavioral therapy (TF-CBT), mindfulness-based interventions, cognitive rehabilitation strategies, and biofeedback/neurofeedback. *Results*: Across study designs, trauma exposure and PTSD in youth are consistently linked to impairments in attentional control and executive functioning, with downstream effects on everyday memory and academic performance. Neurobiological studies commonly implicate altered reactivity within amygdala-centered threat circuits and reduced top-down modulation by prefrontal networks, although findings vary with trauma type, developmental stage, and comorbidity. TF-CBT remains the best-supported intervention for pediatric PTSD symptoms; however, neurocognitive outcomes are measured less frequently. Mindfulness-based programs show promise for strengthening attention and emotion regulation when carefully adapted for trauma-exposed youth. Neurofeedback and targeted cognitive rehabilitation represent emerging approaches with preliminary evidence, but the literature remains heterogeneous. *Conclusions*: An intervention strategy that combines symptom-focused trauma therapy with explicit targeting of executive control, memory processes, and affect regulation may represent a developmentally informed clinical framework for trauma-exposed youth. Future trials need to incorporate standardized neuropsychological endpoints and examine moderators that inform treatment matching.

## 1. Introduction

Exposure to potentially traumatic events in childhood and adolescence, including interpersonal violence, maltreatment, accidents, disasters, and community violence, might precipitate a range of psychological responses. While many young people show resilience, a subset develop persistent posttraumatic stress symptoms and broader internalizing or externalizing difficulties [1,2]. In developmental terms, trauma could disrupt the acquisition of regulatory capacities and executive skills that typically mature across childhood and adolescence, thereby increasing vulnerability to later psychopathology [3,4].

Posttraumatic stress disorder (PTSD) is characterized by re-experiencing, avoidance, negative alterations in cognition and mood, and hyperarousal, and commonly co-occurs with depressive and anxiety disorders [5]. Importantly, the burden of PTSD in youth extends beyond emotional distress. Converging evidence suggests that trauma exposure and PTSD are associated with measurable changes in cognitive functioning, in attention, working memory, processing speed, and executive control, domains that are central to academic achievement and social development [5,6,7].

Neurobiological models highlight alterations within fronto-limbic circuitry. Heightened responsivity of the amygdala to threat cues and diminished regulatory engagement of the prefrontal cortex might contribute to persistent hypervigilance, irritability, and difficulties in emotion regulation [8,9]. At the same time, neuroplasticity, the capacity of neural systems to adapt in response to experience, offers a plausible mechanism through which early, developmentally attuned interventions can support recovery and restore functional trajectories.

The present review has three specific aims: (1) to synthesize evidence on the neuropsychological sequelae associated with trauma exposure and posttraumatic stress disorder (PTSD) in children and adolescents; (2) to critically examine intervention approaches that may influence neurocognitive and affective functioning, including trauma-focused cognitive behavioral therapy (TF-CBT), mindfulness-based interventions, cognitive rehabilitation strategies, and neurofeedback; and (3) to identify clinically relevant implications and priorities for future research. A neuropsychological perspective is particularly valuable in pediatric populations, as trauma-related alterations in attention, executive functioning, and memory may directly interfere with developmental trajectories, learning processes, and engagement with treatment, extending beyond symptom-based conceptualizations of PTSD.

Based on these considerations, this review also outlines key priorities for future research, including the need for standardized neuropsychological endpoints, developmentally sensitive intervention designs, and implementation-focused studies across diverse contexts. To address these aims, a structured narrative review methodology was employed.

## 2. Materials and Methods

This study was conducted as a structured narrative review using a transparent but non-systematic literature search approach. This approach is particularly appropriate given the heterogeneity of study designs and outcome measures in the pediatric PTSD literature. A structured search was performed across multiple electronic databases, including PubMed, Scopus, PsycINFO, Embase, EBSCOhost, Web of Science, and Google Scholar (USA). The search covered publications from January 1990 to March 2025 and was limited to English-language studies involving human participants aged 3–18 years. The review aimed to provide a broad and clinically relevant synthesis of neuropsychological outcomes associated with trauma exposure and posttraumatic stress disorder (PTSD) in youth, as well as intervention approaches targeting neurocognitive and affective functioning.

The search strategy employed combinations of keywords related to trauma, PTSD, neuropsychological functioning, and intervention approaches. Representative search terms included: (“PTSD” OR “trauma”) AND (“executive function” OR “working memory” OR “attention”) AND (“children” OR “adolescents”), as well as terms related to intervention modalities such as “cognitive rehabilitation,” “mindfulness,” “neurofeedback,” and “treatment.” Search terms were adapted as appropriate for each database. In addition, reference lists of relevant articles were manually screened to identify further studies of potential relevance. Given the narrative nature of the review, a formal Boolean search strategy was not exhaustively predefined; instead, the search process was iterative and refined to ensure comprehensive coverage of key domains.

Study selection was guided by predefined criteria to ensure relevance to the aims of the review. Studies were considered eligible if they: (a) included children or adolescents (aged 3–18 years); (b) examined neuropsychological outcomes associated with trauma exposure or PTSD and/or evaluated interventions targeting cognitive or affective functioning; and (c) were published in English between 1990 and 2025. Studies focusing exclusively on adult populations, lacking neurocognitive or functional outcomes, or consisting solely of case reports or non-empirical publications were not considered. Studies were identified and selected through an iterative and concept-driven process, guided by relevance to the aims of the review rather than a formal screening protocol. Studies were appraised qualitatively based on key methodological characteristics, including study design, sample size, and consistency of findings, without the use of a standardized risk-of-bias tool. The overall synthesis should therefore be interpreted as a structured narrative integration of the literature, prioritizing conceptual coherence, clinical relevance, and developmental considerations. Accordingly, the present review should be interpreted as a structured narrative synthesis that prioritizes conceptual integration and clinical relevance, rather than as a fully systematic review. Although predefined criteria guided study selection, the process remained flexible and concept-driven, consistent with a structured narrative review design. No attempt was made to generate an exhaustive or reproducible dataset of included studies; instead, the emphasis was placed on identifying representative and conceptually informative research. Accordingly, the present review should be interpreted as a structured narrative synthesis rather than a systematic review or formal evidence appraisal.

## 3. Results

### 3.1. Neuropsychological Outcomes Following Trauma Exposure and PTSD

Trauma exposure and posttraumatic stress disorder (PTSD) in children and adolescents are associated with a range of neuropsychological vulnerabilities that may interfere with learning, emotional regulation, and adaptive functioning. These effects are not uniform but tend to cluster across core cognitive domains. For clarity, neuropsychological outcomes are organized below according to major domains of functioning.

#### 3.1.1. Attention and Processing Speed

Difficulties in attentional control and processing speed are among the most consistently reported neuropsychological sequelae of trauma exposure in youth. Children with PTSD frequently exhibit impairments in sustained attention, increased distractibility, and reduced capacity to filter irrelevant stimuli, which may reflect heightened threat monitoring and hypervigilance [5,10]. These attentional disruptions can compromise classroom engagement and the ability to maintain goal-directed behavior over time.

Processing speed may also be reduced, particularly in the presence of heightened emotional arousal or comorbid affective symptoms. Slower cognitive processing can further exacerbate difficulties in academic performance and daily functioning, especially in environments that demand rapid information integration. From a neurobiological perspective, such impairments may be linked to altered frontal–limbic interactions, where increased amygdala reactivity interferes with efficient top-down regulation by prefrontal networks.

However, findings should be interpreted cautiously, as many studies rely on small clinical samples and cross-sectional designs, with limited control for confounding variables such as sleep disturbance, medication use, and socioeconomic adversity.

#### 3.1.2. Working Memory

Working memory, a core component of executive functioning that supports the temporary storage and manipulation of information, is also frequently affected in trauma-exposed youth. Impairments in working memory may manifest as difficulties following multi-step instructions, organizing information, and maintaining task-relevant goals in the presence of distraction.

These deficits are particularly salient in educational contexts, where working memory capacity underpins reading comprehension, problem-solving, and learning efficiency. Neurobiologically, disruptions in dorsal prefrontal systems implicated in working memory processes may contribute to these observed difficulties, especially under conditions of emotional stress or heightened arousal.

Nevertheless, the evidence base remains heterogeneous, with variability in the assessment tools used and inconsistent reporting of effect sizes. In addition, the contribution of comorbid conditions, such as depression or attention-deficit/hyperactivity disorder (ADHD), is not always adequately controlled, limiting causal interpretation.

#### 3.1.3. Executive Function

Executive functioning appears to be one of the most consistently affected domains in children with trauma exposure and PTSD. Deficits have been documented across multiple components, including inhibitory control, cognitive flexibility, planning, and set-shifting [6,11]. For example, maltreated children with PTSD have demonstrated poorer performance on tasks such as the Stroop Color–Word Test and the Wisconsin Card Sorting Test, indicating difficulties in interference control and flexible adaptation to changing task demands.

Clinically, these impairments may manifest as impulsivity, difficulty regulating behavior, and reduced capacity to shift attention away from trauma-related cues. Such executive dysfunction can interfere not only with academic functioning but also with engagement in psychotherapy, where sustained attention, cognitive flexibility, and emotional regulation are required.

However, it is important to note that executive deficits in trauma-exposed youth may reflect both trait vulnerabilities and state-dependent effects related to hyperarousal or emotional distress. The lack of longitudinal studies and standardized neuropsychological batteries further limits the ability to determine the persistence and specificity of these impairments.

#### 3.1.4. Memory Function

Memory disturbances in trauma-exposed youth represent a complex and multifaceted domain, encompassing both everyday memory deficits and alterations in the encoding and retrieval of emotionally salient information. Empirical studies have shown that children with PTSD may perform more poorly on measures of everyday memory, such as the Rivermead Behavioural Memory Test, compared with trauma-exposed peers without PTSD [12].

In addition to deficits in deliberate recall, trauma is associated with qualitative changes in memory processing, including the presence of intrusive memories and, in some cases, fragmented or incomplete recall of traumatic events. These patterns may reflect the interaction between heightened emotional arousal, attentional narrowing during encoding, and subsequent avoidance processes.

Neurodevelopmental evidence further suggests that early adversity may be associated with alterations in white matter integrity, which in turn correlate with cognitive performance [7], providing a plausible biological pathway linking trauma exposure to memory-related outcomes.

Despite these findings, the literature is characterized by substantial variability in how memory is assessed, with limited differentiation between verbal, visual, and autobiographical memory systems. Moreover, few studies systematically examine how memory processes change following intervention, representing a key gap in the field.

Overall, while evidence supports the presence of neuropsychological vulnerabilities across multiple domains, the heterogeneity of study designs, populations, and measurement approaches underscores the need for more standardized, longitudinal, and developmentally sensitive research.

### 3.2. Neurobiological Correlates and Neural Systems Implicated in Pediatric PTSD

Neurobiological accounts of PTSD emphasize alterations in circuits subserving threat detection, learning, and top-down regulation. The amygdala plays a central role in detecting salient cues and orchestrating fear responses; heightened amygdala reactivity has been observed in PTSD and might be conceptualized as a disruption in fear learning and extinction processes [8,13]. The amygdala’s functional coupling with the hippocampus and medial prefrontal cortex is relevant for contextualizing threat and regulating conditioned fear responses [9].

In parallel, reduced recruitment of prefrontal networks involved in cognitive control and emotion regulation might contribute to difficulties inhibiting trauma-related responses and to heightened reactivity in the face of reminders [14,15]. Disruptions in dorsal prefrontal networks might be expressed clinically as slower processing, compromised working memory, and impaired flexible control of attention, features that can reinforce avoidance and perpetuate symptom maintenance [16]. Although the broader PTSD neurocircuitry literature is extensive, pediatric findings remain heterogeneous, highlighting the need for developmentally sensitive designs and careful control of comorbidities and medication effects.

However, findings should be interpreted cautiously, as many studies are characterized by small sample sizes, cross-sectional designs, and limited control for confounding variables such as comorbid conditions and socioeconomic adversity.

### 3.3. Memory Processing in the Aftermath of Trauma

Traumatic events might shape memory in two clinically important ways: by fostering intrusive re-experiencing and by disrupting deliberate, coherent recall. Intrusive memories are common in the acute aftermath of trauma and can be highly distressing, interfere with concentration, and provoke avoidance [17,18]. At the same time, intrusive recollections might serve adaptive functions, such as signaling potential danger or helping to preserve the continuity of autobiographical memory [19,20].

Conversely, some trauma-exposed individuals report partial amnesia or fragmented recall for elements of the event. Such variability underlines that memory disturbances in PTSD reflect the interplay of arousal, attentional narrowing, and subsequent meaning-making [21]. Clinically, the timing and quality of post-trauma memory processing are relevant because they inform early intervention strategies and guide therapeutic work on trauma narratives.

### 3.4. Emotional Reactivity and Emotion Regulation

Difficulty regulating negative affect is a core feature of posttraumatic stress responses and is linked to both symptom severity and functional impairment. A pattern of heightened emotional reactivity, often conceptualized as amygdala-driven threat sensitivity coupled with diminished prefrontal modulation, could manifest as irritability, hyperarousal, and a reduced threshold for distress [22,23]).

Emotion regulation difficulties might be amplified by secondary emotions such as shame, guilt, and anger, which constrain disclosure and impede engagement with treatment [24]. Non-acceptance of negative emotions might further intensify distress by adding layers of self-criticism or avoidance, thereby limiting access to adaptive coping behaviors [16]. In some youth, trauma-related difficulties in identifying and describing emotions (secondary alexithymia) might function as an avoidant strategy, reinforcing posttraumatic symptom persistence [16]. These observations support the clinical emphasis on interventions that strengthen affect labeling, distress tolerance, and flexible regulation strategies.

### 3.5. Intervention Approaches Targeting Neuropsychological Functioning

Where available, emphasis was placed on evidence derived from randomized controlled trials and longitudinal studies in pediatric populations; however, given the limited and heterogeneous nature of the literature, some conclusions necessarily draw on broader evidence, including adult PTSD and related neurorehabilitation research, which are explicitly interpreted with caution. Interventions for pediatric PTSD have historically prioritized symptom reduction. However, given evidence of neurocognitive vulnerabilities, it needs to be considered whether and how treatments modify attention, executive control, memory processes, and emotion regulation. Below, we summarize evidence for established and emerging approaches, with an emphasis on neuropsychological targets and clinical implementation.

#### 3.5.1. Cognitive Rehabilitation and Compensatory Strategies

Cognitive rehabilitation refers to structured interventions designed to restore or compensate for cognitive impairments. Although the strongest evidence base derives from neurorehabilitation contexts, several principles are relevant to trauma-exposed youth, when PTSD co-occurs with attentional or executive difficulties. Core components include collaborative goal setting, task decomposition, and prioritization strategies that reduce cognitive load in daily routines [25,26].

Memory supports like external aids, environmental structuring, and mnemonic techniques, facilitate learning and everyday functioning when working memory and prospective memory are compromised [20]. In clinical practice, cognitive rehabilitation strategies might be most useful when integrated with trauma-focused psychotherapy, ensuring that cognitive supports enhance, rather than replace, processing of trauma-related material.

#### 3.5.2. Biofeedback and Neurofeedback

Biofeedback and neurofeedback provide individuals with real-time information about physiological or neural signals, with the aim of enhancing self-regulatory capacity. In PTSD, these approaches have been proposed as methods to modulate arousal and improve cognitive control [27].

Emerging randomized trials suggest that neurofeedback might influence neural systems implicated in emotional and cognitive control. For example, alpha-based neurofeedback has been associated with increased top-down modulation during symptom provocation working memory tasks [28]. A feasibility study in refugees with chronic, treatment-resistant PTSD also reported improvements in cognitive control following neurofeedback therapy [29]. While encouraging, the pediatric evidence base remains limited, and future work need to clarify optimal protocols, developmental considerations, and clinically meaningful cognitive endpoints.

#### 3.5.3. Mindfulness-Based Interventions

Mindfulness-based interventions aim to cultivate nonjudgmental attention to present-moment experience and have been applied across a range of psychiatric conditions, including depression and anxiety [30]. In trauma-exposed youth, mindfulness might support attentional control, improve emotion regulation, and reduce impulsive responding [31,32].

Mindfulness-Based Stress Reduction (MBSR) is typically delivered as an eight-week program combining meditation practices and psychoeducation. In a pilot randomized trial with traumatized youth in foster care, MBSR-related techniques were associated with reductions in stress and improvements in social functioning [33]. Additional work in outpatient and school-adjacent settings suggests feasibility and potential benefits for coping skills, self-awareness, and stress reduction [34,35,36].

Mindfulness-Based Cognitive Therapy (MBCT) integrates mindfulness with cognitive behavioral techniques aimed at disrupting perseverative negative thinking. Systematic work suggests that mindfulness-based approaches might influence neuroplasticity in networks supporting emotion regulation, potentially attenuating amygdala reactivity while strengthening prefrontal engagement [37]. Nonetheless, mindfulness might initially intensify distress in some trauma-exposed individuals by increasing contact with traumatic memories; careful pacing, containment strategies, and clinician expertise are therefore essential [38,39].

#### 3.5.4. Trauma-Focused Cognitive Behavioral Therapy

Trauma-Focused Cognitive Behavioral Therapy (TF-CBT) is the most extensively studied intervention for pediatric PTSD and is supported by a substantial body of randomized and quasi-experimental evidence [40,41,42]. TF-CBT integrates psychoeducation, skills training, gradual exposure, and caregiver involvement within a phased treatment model. The well-known PRACTICE components, e.g., parenting and psychoeducation, relaxation, affect expression and modulation, cognitive coping, trauma narration and processing, In vivo mastery, Conjoint sessions, and Enhancing safety and future development, provide a coherent structure for addressing both symptom reduction and skill development [43].

TF-CBT is typically delivered over 12–20 sessions (60–90 min each) to youth aged approximately 3–18 years, with parallel caregiver sessions to strengthen support and parenting practices [40]. Trials in diverse populations, including youth exposed to sexual abuse, community violence, war-related trauma, and disasters, have generally demonstrated improvements in PTSD symptoms and related internalizing problems, with gains often maintained at follow-up [44,45,46,47,48].

From a neuropsychological standpoint, TF-CBT might indirectly support cognitive functioning by reducing hyperarousal and avoidance, thereby increasing capacity for sustained attention and learning. However, neurocognitive outcomes are infrequently assessed, limiting conclusions about direct effects on executive function or memory. Moreover, clinical complexity matters when severe behavioral dysregulation, active suicidality, or substance use is prominent, trauma exposure components might require careful sequencing or adjunctive stabilization strategies [42]. Implementation in schools also depends on organizational readiness and provider-level factors, including attitudes toward evidence-based practice [49,50,51].

Across studies, methodological heterogeneity, variability in neuropsychological measures, and limited longitudinal designs constrain the strength of conclusions. Few studies directly assess changes in neurocognitive outcomes following intervention, and effect sizes are rarely reported, limiting the ability to determine clinical significance.

Tables are intended to provide a structured overview of intervention characteristics and should be interpreted as descriptive summaries rather than formal evidence grading. To facilitate clinical translation, Table 1 summarizes the principal intervention models discussed in this review, mapping each approach to its putative neuropsychological targets, typical delivery parameters, and the strength and limitations of the pediatric evidence base. Importantly, ‘evidence strength’ below reflects the quality and quantity of studies evaluating PTSD symptom outcomes and/or neurocognitive outcomes in young populations, without implying interchangeability across trauma types, developmental stages, comorbidity patterns, or service contexts [27,30,37,40,52]. The categorization of evidence presented in the tables reflects an indicative level of support based on the available literature and should not be interpreted as a formal grading of evidence quality. These tables are intended as integrative, conceptually driven summaries and do not constitute formal evidence grading or meta-analytic synthesis.

Rather than offering a descriptive listing of studies, Table 2 synthesizes the literature according to intervention approach and neuropsychological targets, highlighting convergences and divergences across study designs. The table is organized to reflect a developmental and neuropsychological framework, linking intervention types to the cognitive and emotional processes they primarily target, rather than focusing solely on symptom reduction. The reported narrative level of support reflects a qualitative and conceptually driven synthesis of the literature rather than formal evidence appraisal.

The categorization reflects a qualitative, non-systematic appraisal based on frequency, consistency, and type of available studies, and should not be interpreted as formal evidence grading.

The following table summarizes neurocognitive domains and their associated intervention targets in a descriptive and conceptually oriented manner (Table 3).

## 4. Discussion

This review highlights that pediatric PTSD and trauma exposure are best conceptualized not only as disorders of emotion and memory, but also as conditions frequently involving disruption in neurocognitive systems that support attention, executive functioning, and learning. These cognitive vulnerabilities are clinically meaningful, as they may shape educational outcomes, social functioning, and the capacity to engage effectively in psychotherapy [6,12]. Neurobiological models emphasizing fronto-limbic dysregulation provide a coherent explanatory framework, linking heightened threat sensitivity with reduced top–down regulatory control [9].

From a clinical standpoint, these findings support a more integrative assessment approach that extends beyond symptom-based evaluation to include developmental history, functional performance in educational settings, and, where feasible, targeted neuropsychological assessment. Identifying impairments in domains such as executive functioning or working memory may inform treatment pacing, the selection of therapeutic techniques, and the need for compensatory supports across home and school environments.

Intervention planning should therefore adopt a developmentally informed and functionally oriented perspective. Trauma-focused approaches, particularly TF-CBT, remain the first-line treatment for symptom reduction, while adjunctive strategies may be indicated when neurocognitive vulnerabilities interfere with engagement or functional recovery. Cognitive rehabilitation techniques may provide structured support for attention and executive skills in daily contexts, whereas mindfulness-based interventions and neurofeedback approaches may contribute to improved regulation of attention and emotional arousal, when appropriately adapted for developmental level and trauma sensitivity [27,28,30].

Clinical complexity, including comorbidity, sleep disruption, and socio-environmental stressors, further underscores the need for individualized and flexible intervention planning. In particular, overlapping symptom presentations (e.g., PTSD and ADHD-like attentional difficulties) require careful differential formulation to avoid misinterpretation of cognitive profiles and to guide appropriate sequencing of interventions [6,53,54]. Stabilization strategies targeting arousal and sleep may be necessary precursors to effective trauma processing, while caregiver involvement and contextual supports remain essential components of intervention [55].

Overall, the integration of neuropsychological and clinical perspectives offers a more comprehensive framework for understanding pediatric PTSD and for tailoring interventions to support both symptom reduction and functional recovery. By explicitly linking neurocognitive domains with intervention mechanisms, this review contributes a clinically applicable framework that extends beyond symptom-focused models of pediatric PTSD.

### Limitations

This review has several methodological limitations that should be considered when interpreting the findings. First, although a structured search strategy was employed, the review is narrative in nature and does not include a formal risk-of-bias assessment, which may limit the ability to evaluate the quality of included studies systematically. Second, the literature on pediatric PTSD is characterized by substantial heterogeneity in study design, sample characteristics, and measurement approaches, which constrains comparability across studies and limits the strength of inferences.

Third, many studies do not adequately control for key confounding variables, including socioeconomic adversity, sleep disturbance, medication use, and comorbid psychiatric or neurodevelopmental conditions. These factors may significantly influence both neuropsychological functioning and treatment outcomes. Additionally, variability in the assessment of cognitive domains, particularly executive functioning, working memory, and attention, further complicates synthesis and interpretation of findings.

Publication bias also represents a potential limitation, as studies reporting positive outcomes are more likely to be published. Finally, the diversity of trauma types and developmental stages represented in the literature limits generalizability, as different forms of trauma exposure may be associated with distinct neurodevelopmental trajectories and cognitive profiles.

## 5. Conclusions

Trauma exposure and PTSD in youth are associated with clinically significant vulnerabilities in attention, executive functioning, memory processing, and emotion regulation, with important implications for learning, social development, and treatment engagement. TF-CBT remains the most robustly supported intervention for pediatric PTSD symptoms, while adjunctive approaches, including mindfulness-based interventions, neurofeedback, and cognitive rehabilitation strategies, may offer additional benefits when neurocognitive difficulties are present.

A developmentally informed and neuropsychologically attuned approach, integrating trauma-focused therapy with strategies that support cognitive and regulatory functioning, represents a clinically plausible and conceptually informed framework. The conclusions of this review should be interpreted in light of its narrative design, which prioritizes conceptual integration over systematic evidence synthesis.

Future research should prioritize the use of standardized neuropsychological outcome measures to better understand treatment effects on cognitive functioning.

## Figures and Tables

**Table 1:** Intervention approaches for trauma-exposed youth; putative neuropsychological targets, delivery characteristics, evidence base, and practical constraints.

Intervention Approach	Putative Neuropsychological Targets (Typical Emphasis)	Typical Delivery Parameters (Illustrative)	Evidence Base in Youth with PTSD	Practical Constraints and Clinical Notes
Trauma-Focused CBT (TF-CBT)	Threat-linked emotion regulation; cognitive coping/reappraisal; trauma-memory integration; reduced avoidance/hyperarousal; caregiver scaffolding of regulation	Manualized, phased (skills/stabilization to narration/processing to consolidation); often 12–20 sessions, 60–90 min; child & caregiver components	Most extensively studied for symptom outcomes	Requires readiness for trauma processing; consider sequencing/stabilization first when severe externalizing, active suicidality, or substance use compromises safety/engagement; school implementation can be constrained by organizational/provider factors
Mindfulness-based interventions (MBSR/MBCT; mindfulness skills)	Attentional control; reduced cue-reactivity; distress tolerance/interoceptive awareness; strengthened top-down modulation of affect	Often group-based; MBSR classically 8 weeks with home practice; dose varies across pediatric adaptations	Preliminary and heterogeneous evidence	Trauma-related intrusions or distress can intensify early if pacing is not carefully titrated; outcomes depend on developmental capacity, adherence, and trauma-informed delivery
Cognitive rehabilitation/compensatory strategies	Attention/processing speed; working memory efficiency; executive skills (planning, organization, flexibility); academic and everyday functioning via compensatory supports	Individualized strategy training (goal management, graded practice, metacognitive coaching, external aids) & coordination with school/home; intensity variable	Limited PTSD-specific evidence; strong conceptual rationale for functional support	Attribution can be difficult when sleep disruption, mood symptoms, or environmental instability are active; cognitive supports may improve functioning yet not resolve the PTSD intrusion–avoidance cycle without trauma-focused work
Biofeedback/Neurofeedback	Arousal regulation; autonomic stability; improved cognitive control under emotional load; modulation of threat responsivity	Multi-session clinic protocols (EEG-based or peripheral measures); frequently adjunctive to psychotherapy	Emerging, protocol heterogeneity; pediatric evidence growing but uneven	Access/cost/training constrain scalability; protocol variability complicates synthesis; best conceptualized as adjunctive unless trauma meaning-making/avoidance are also addressed

**Table 2:** Critical Evaluation of Intervention Evidence for Neuropsychological and Clinical Outcomes in Pediatric PTSD.

Intervention Type	Target Population	Study Designs Included	Neuropsychological Targets	Key Outcomes Reported	Narrative Level of Support (Symptoms vs. Neurocognition)	Main Consistency Issues Across Studies	Key Limitations/Principal Methodological Limitations
Cognitive Rehabilitation	Children/adolescents with trauma exposure or PTSD	Small RCTs; longitudinal; clinical intervention studies	Attention, working memory, processing speed, executive functioning	Improvements in attention, compensatory memory strategies, task organization, functional skills	Symptoms: Limited/indirect Neurocognition: Moderate but inconsistent	Moderate heterogeneity; limited replication; small samples	Non-standardized protocols; variability in intervention intensity; inconsistent outcome measures; limited ecological validity
Mindfulness-Based Interventions (MBSR, MBCT)	Trauma-exposed youth (incl. foster care, outpatient populations)	RCTs; pilot studies; mixed-methods	Attention regulation, emotional awareness, stress reactivity	Reduced anxiety/depression; improved emotion regulation and social functioning	Symptoms: Moderate Neurocognition: Emerging/preliminary	Variable methodological rigor; some replication but small samples	Risk of initial distress activation; selection bias; adherence variability; limited long-term follow-up
Trauma-Focused Cognitive Behavioral Therapy (TF-CBT)	Children/adolescents (3–18) with PTSD	Multiple high-quality RCTs; longitudinal follow-ups; cross-cultural studies	Emotion regulation, trauma processing, cognitive restructuring, behavioral control	Robust reductions in PTSD, anxiety, depression; improved coping and caregiver functioning	Symptoms: Strong Neurocognition: Limited/under-measured	High consistency across settings and populations; strong replication	Neurocognitive outcomes rarely assessed; possible performance bias; requires adaptation for complex comorbidity
Integrated/Multimodal Approaches	Youth with complex trauma and comorbidity	Comparative trials; implementation studies	Combined cognitive, emotional, behavioral domains	Broad improvements in functioning; potential synergistic effects	Symptoms: Emerging Neurocognition: Emerging/unclear	Low-to-moderate consistency; heterogeneous designs	Lack of standardized frameworks; difficulty isolating active components; limited scalability data; weak causal inference

**Table 3:** Mapping Neurocognitive Domains, Mechanistic Systems, and Intervention Targets in Pediatric PTSD.

Neurocognitive Domain	Key Subcomponents	Associated Neurobiological Systems	Primary Intervention Targets	Intervention Types	Narrative Indication of Cognitive Change Evidence
Attention	Sustained, selective attention	Prefrontal cortex; frontoparietal networks; HPA-axis modulation	Attentional control, arousal regulation	Mindfulness-based interventions; cognitive rehabilitation; neurofeedback	Emerging (moderate for mindfulness on attention regulation; limited for cognitive remediation)
Working Memory	Verbal, visuospatial maintenance and manipulation	Dorsolateral prefrontal cortex; hippocampal–prefrontal circuits	Cognitive load management; information updating	Cognitive rehabilitation; TF-CBT (indirect support)	Emerging to moderate (limited RCT support for direct training effects)
Processing Speed	Cognitive efficiency, response latency	Distributed white matter networks; frontostriatal pathways	Cognitive efficiency, task performance speed	Cognitive rehabilitation (limited)	Limited/preliminary evidence
Executive Function	Inhibition, cognitive flexibility, planning	Prefrontal cortex; anterior cingulate cortex	Top-down cognitive control, behavioral regulation	TF-CBT; cognitive rehabilitation	Moderate for TF-CBT (indirect); emerging for cognitive training
Learning & Memory	Encoding, consolidation, retrieval	Hippocampus; medial temporal lobe networks	Trauma narrative processing; memory integration	TF-CBT; cognitive rehabilitation (experimental)	Moderate for TF-CBT (symptom-linked); limited for cognitive change
Affect Regulation	Emotion recognition, modulation, stress response	Amygdala–prefrontal circuitry; HPA axis	Emotion regulation; threat reactivity reduction	TF-CBT; mindfulness-based interventions	Strong for TF-CBT (symptoms); moderate for mindfulness

## Data Availability

No new data were created or analyzed in this study.
